# The Effect of *Helicobacter pylori* Gene Combinations of *cagA*, *cagE*, *virB11*, *vacA*, and *babA* on the Outcome of Gastric Disease in a Southern Moroccan Population

**DOI:** 10.3390/pathogens14030279

**Published:** 2025-03-14

**Authors:** Mariama Barhoine, Fatima Moustaoui, Omayma Hammani, Mohamed Aghrouch, Zohra Lemkhente, Zineb Belhabib, Zineb Bajaddoub, Anass Touyar, Nourdin Aqoudad, Bouchra Rherissi, Nadia El Kadmiri, Youssef Idaghdour, Fatima Boubrik, Ahmed Belmouden

**Affiliations:** 1 Cell Biology and Molecular Genetics Laboratory, Faculty of Sciences, Ibnou Zohr University, Agadir 80000, Morocco; mariama.barhoine@edu.uiz.ac.ma (M.B.); f.boubrik@uiz.ac.ma (F.B.); 2 Medical Analysis Laboratory, Regional Hospital Centre Hassan II, Agadir 80000, Morocco; 3 Medical-Surgical, Biomedicine and Infectiology Laboratory, Faculty of Medicine and Pharmacy, Ibnou Zohr University, Agadir 80000, Morocco; z.lemkhente@uiz.ac.ma; 4 Gastroenterology Practice, Agadir 80000, Morocco; 5 Gastroenterology Unit, Mokhtar Soussi Hospital, Taroudant 83000, Morocco; 6 Gastroenterology Unit, Hassan II Hospital, Agadir 80000, Morocco; 7 Faculty of Medicine and Pharmacy of Agadir, Ibnou Zohr University, Agadir 80000, Morocco; 8 Geo-Bio-Environment Engineering and Innovation Laboratory, Polydisciplinary Faculty, Ibnou Zohr University, Taroudant 83000, Morocco; 9 Center for Genomics and Systems Biology, New York University Abu Dhabi, Abu Dhabi P.O. Box 129188, United Arab Emirates; youssef.idaghdour@nyu.edu

**Keywords:** *Helicobacter pylori*, virulence genes, gastric disease, Morocco

## Abstract

*Helicobacter pylori* (*H. pylori*) possess an arsenal of virulence genes that makes them the main etiological factor in gastric diseases. In this study, 120 southern Moroccan patients who were dyspeptic were profiled to investigate the potential association between disease severity and the combination of multiple virulence genes. Gastric biopsies were taken from patients, followed by histopathological evaluation and genotyping of *H. pylori* using PCR. *H. pylori* was detected in 58.3% of cases, and genotypes were distributed as follows: *oipA* (94.3%), *cagA* (62.9%), *virB11* (60%), *babA* (55.7%), *dupA* (54.3%), *cagE* (51.4%), *iceA1* (31.4%), *iceA2* (45.7%), *vacA s2m2* (47.1%), *vacA s1m1* (30%), and *vacA s1m2* (7.1%). Statistically significant associations with males were observed for the *cagA*, *cagE*, and *virB11* genes and multiple strain infections. Multivariate analysis revealed an association between *cagE* and heightened neutrophil activity, with an odds ratio (OR) of 4.99 (*p* = 0.03). The gene combination [*cagA* (+), *cagE* (+), *virB11* (+), *vacA s1m1*, and *babA* (+)] emerged as a predictive factor for gastric cancer (OR = 11.10, *p* = 0.046), while the combination [*cagA* (-), *cagE* (-), *virB11* (-), *vacA s2m2*, *babA* (+)] was associated with gastric atrophy (OR = 10.25, *p* = 0.016). Age (≤40 years) (OR = 5.87, *p* = 0.013) and moderate to severe bacterial density (OR = 15.38, *p* = 0.017) were identified as predictive factors for follicular gastritis. These findings underscore the significance of multigene profiling as a prognostic marker and emphasize the importance of age and sex in preventing adverse outcomes in severe gastric diseases.

## 1. Introduction

*H. pylori* colonize the gastric mucosa of more than half the world’s population. This microorganism has been classified as a Group I human carcinogen by the International Agency for Research on Cancer due to its close association with adenocarcinoma and mucosa-associated lymphoma (MALT). The acquisition of this infection typically occurs during early childhood and can persist asymptomatically [1]. However, in approximately 15–20% of infected individuals, the ensuing chronic inflammation initiates chronic active gastritis. This can advance to peptic ulcer disease or, following Correa’s precancerous cascade, progress to adenocarcinoma (non-atrophic gastritis → atrophic gastritis → intestinal metaplasia → dysplasia → adenocarcinoma). Alternatively, it may lead to MALT lymphoma, as outlined in Wotherspoon’s cascade (follicular gastritis → suspicious inflammatory infiltrate, probably reactive → suspicious infiltrate, probably lymphoma → MALT lymphoma) [2,3]. The outcome of the infection depends on strain genotype, host genetic diversity, and environmental factors [4]. *H. pylori* carry an arsenal of virulence genes that have gained considerable momentum due to their medical value as biomarkers of clinical outcome [5]. Urease encoded by *ureABIEFGH* genes neutralizes gastric acidity and modulates the immune response [3,6]. The presence of BabA (blood group antigen–binding adhesin) facilitates the interaction with Lewis b (Leb) antigens expressed on the surface of gastric cells, and infection with BabA-positive strains has been associated with an increased likelihood of developing peptic ulcer disease and gastric cancer [6,7]. The gene induced by contact with the epithelium (*iceA*) consists of *iceA1* and *iceA2*. Expression of *iceA1* is upregulated by contact with epithelial cells, which is linked to increased production of interleukin-8 (IL-8) and inflammation [8]. On the other hand, studies have revealed the role of genes encoding duodenal ulcer promotion protein A (dupA) and outer inflammatory protein A (oipA) in promoting IL-8 secretion [9,10]. *H. pylori* adhesin A (HpaA) and neutrophil activator protein A (NapA) are present in almost all strains and play a critical role as highly immunogenic proteins [11]. The vacuolating cytotoxin A gene (*vacA*), along with the *cagPAI*, constitutes the main virulence factors of *H. pylori*. The *vacA* gene is present in nearly all strains, but the cytotoxic activity of the VacA protein is affected by polymorphisms in three regions: the signal region (s) (s1 or s2 allele), the intermediate region (i) (i1, i2, or i3), and the middle region (m) (m1 or m2). Strains with the *vacA s1m1* combination are associated with high vacuolization activity, while the combinations of *vacA s1m2* and *vacAs2m2* exhibit moderate and minimal to absent activity, respectively [4,12,13]. The *cagPAI* code for both the CagA protein and its type IV secretion system (T4SS). The *cagE*, *virB11* (also known as *cagα*) and *cagβ* are genes encoding ATPase components of the T4SS, which facilitate the translocation of CagA into host cells and contribute to the carcinogenesis process [14,15].

In this study, we report on the prevalence of *H. pylori* infection in the south of Morocco, assess the genetic diversity of eight virulence genes, and investigate, for the first time in North Africa, the association of this relatively large number of virulence genes (*cagA*, *cagE*, *virB11*, *vacA*, *dupA*, *oipA*, *babA*, and *iceA*) and demographic factors with the severity of gastric disease. The results underline the power of combinatorial testing in identifying patients at risk of severe gastric disease and warrant similar testing in other populations.

## 2. Materials and Methods

### 2.1. Clinical Specimens

A total of 120 gastric biopsies were obtained between April 2019 and January 2022 from consenting patients who suffered from dyspepsia symptoms and underwent endoscopy at the gastroenterology unit in both private and public clinics located in the cities of Agadir and Taroudant, Morocco. We excluded from this study pregnant women, patients diagnosed with esophageal cancer or liver failure, patients who had experienced gastrointestinal bleeding or undergone surgery, as well as the patients who have received non-steroidal anti-inflammatory drugs, antibiotics, and/or proton pump inhibitors within the preceding two weeks, and patients with incomplete histological results. The socioeconomic and medical history of each patient was collected using questionnaires and medical records.

### 2.2. Histopathological Exams

Histopathological analysis was conducted on biopsies obtained from the antrum, corpus, and angulus, following the Sydney system, by two independent pathologists [16]. To achieve statistical significance, we divided the patients into 2 groups according to their diagnosis: gastritis (*n* = 51; 42.5%) and severe gastric diseases (*n* = 69; 57.5%) that includes follicular gastritis (32; 26.7%), peptic ulcer (4; 3.33%), gastric polyps (1; 0.83%), atrophic gastritis (13; 10.8%), intestinal metaplasia (11; 9.2%), dysplasia (1; 0.83%) and gastric cancer (7; 5.8%) cases. Neutrophil activity, the degree of chronic inflammation and bacterial density were scored as follows: 0, absent; 1, mild; 2, moderate; and 3, severe. For statistical considerations, we classified the parameters of neutrophil activity, chronic inflammation, and bacterial density into 2 categories: absent or mild and moderate or severe. The overall characteristics of the 120 patients are shown in Supplementary Table S1.

### 2.3. DNA Extraction

Genomic DNA was directly extracted from antral and/or fundic gastric biopsies using the QIAamp DNA Mini Kit (QIAGEN, Hilden, Germany) following the manufacturer’s guidelines, then stored at −20 °C until analysis. DNA samples were quantified and quality controlled using the DeNovix DS-11 spectrophotometer (DeNovix Inc., Wilmington, DE, USA). All specimens were tested for DNA integrity and the presence of PCR inhibitors by amplification of the human β-globin gene.

### 2.4. The Detection of H. pylori and Its Virulence Genes

*H. pylori* infection is confirmed by amplification of the *ureA*, *ureB*, *glmM (housekeeping gene*), *hpA*, and *napA* genes. Positive samples were genotyped using primers: *cagA*, *cagA3’v*, *vacA*, *cagE*, *virB11*, *dupA*, *oipA*, *iceA*, and *babA*; as described elsewhere [6,17,18,19,20,21,22], detailed information is listed in Supplementary Table S2. The *cagA* results were considered positive if at least one of the two gene regions was positive. Detection of the *babA* gene was carried out using a forward primer as described by Homan et al. [23] and a reverse primer reported by Gerhard et al. [24] to amplify a 505 bp sequence. Each gene was detected by a single PCR except (*ureA*, *ureB)* and (*hpA*, *napA)*, which were amplified by duplex PCR. All runs included sterile Milli-Q water as a negative control, and a positive control comprised *H. pylori* DNA extracted from a confirmed positive strain culture, verified by Sanger sequencing. Negative results were confirmed by PCR re-testing. The PCR products were visualized using standard agarose gel electrophoresis.

### 2.5. Statistical Analysis

Statistical analysis was performed using the SPSS version 26 software. Bivariate analysis was performed using Chi-square or Fisher’s exact. The strength of association was determined by odds ratios calculated from the contingency tables. A multiple correspondence analysis (MCA) was used to identify the overall relationship between *H. pylori* genes. Multivariate logistic regression was performed to identify potential predictor(s) associated with each histopathological parameter. Variables with *p*-values < 0.10 obtained from bivariate analysis were included in multivariate analysis, and the results were expressed as adjusted odds ratios (ORa) with 95% confidence intervals (CI) and *p*-values. For all tests, *p*-values < 0.05 were considered statistically significant.

## 3. Results

### 3.1. Characteristics of Infected Patients

**Figure 1:**
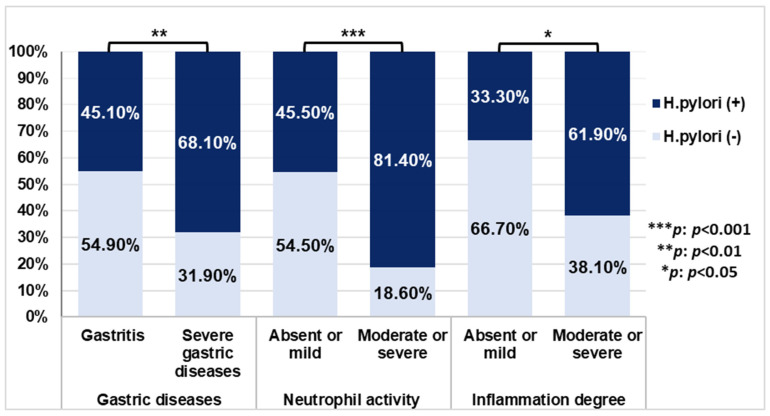
The distribution of *H. pylori* infection in histopathological manifestations. *p*-values were calculated by Chi-square test.

PCR confirmed *H. pylori* infection in 58.3% (70/120) of cases. Regarding the association of infection with demographic risk factors (age, sex, smoking, alcohol consumption, and place of residence), a significant correlation was found between infection and males (*p* = 0.028). *H. pylori* infection was more frequent in younger patients of age ≤40, smokers/ex-smokers, alcohol drinkers, and rural subjects, although the association was not statistically significant (*p* > 0.05) (Supplementary Table S3). The distribution of histopathological findings based on *H. pylori* status showed a significant association of *H. pylori* infection with severe gastric disease, *p* = 0.011 (OR = 2.60; 95% CI: 1.23–5.50), as well as with moderate/severe levels of neutrophil activity and the degree of inflammation *p* < 0.001 (OR = 5.25; 95% CI: 2.16–12.78), and *p*= 0.036 (OR = 3.25; 95 % CI: 1.04–10.20), respectively (Figure 1).

### 3.2. The Distribution of H. pylori Genotypes in Infected Patients

The distribution of virulence genes tested in patients infected with *H. pylori* showed that 94.3% of patients were positive for *oipA*, 62.9% for *cagA*, 60% for *virB11*, 55.7% for *babA*, 54.3% for *dupA* and 51.4% for *cagE*. *iceA1* was detected in 31.4% of patients and *iceA2* in 45.7%, while 15.7% of patients were positive for both *iceA1* and *iceA2*. Regarding the *vacA* gene, all *H. pylori*-positive subjects were *vacA s* (+), and among patients harboring strains with a single *vacA s* allele, the *vacA s2* genotype was predominant (50%) and *vacA s1* accounted for 38.6%. As for the m region in strains carrying a single *vacA m* allele, the *vacA m2* genotype (58.6%) was more prevalent than the *vacA m1* genotype (31.4%), whereas 2.9% of participants carried incomplete *vacA m* strains. The *vacA sm* profile was also studied, and the most frequent was *vacA s2m2* (47.1%), followed by *vacA s1m1* (30%) and *vacA s1m2* (7.1%), while *vacA s2m1* genotype was not detected in our cohort. A total of 24.3% of cases were classified as having multiple infections based on the presence of two alleles of the same gene, either *vacA* or *iceA* or both (Supplementary Table S4).

### 3.3. The Relationship Between H. pylori Virulence Genes

**Figure 2:**
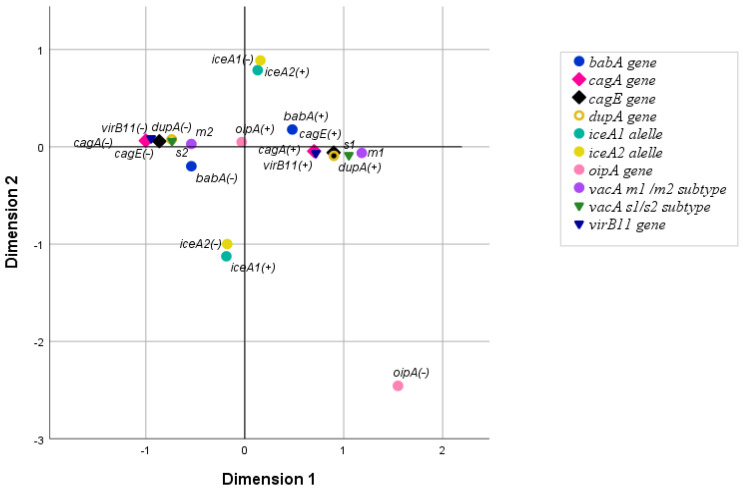
The relationship between *H. pylori* virulence genes, analyzed by MCA.

MCA analysis was conducted to examine the association between the eight studied virulence genes in patients infected with a single strain of *H. pylori* (51/70 patients). The analysis excluded incomplete genotypes and multiple infections. The resulting MCA plot revealed two main clusters on the horizontal axis: cluster 1 included strains with genotypes *cagA* (+), *cagE* (+), *virB11* (+), *vacA s1*, *vacA m1*, *babA* (+), and *dupA* (+), while cluster 2 comprised strains with genotypes *vacA s2*, *vacA m2*, and lacking *cagA*, *cagE*, *virB11*, *babA*, and *dupA* (Figure 2).

The strength of the association between the eight genes was then measured using the Chi-square test or Fisher’s exact test. The genes *cagA*, *cagE*, *virB11*, *vacA s1*, *vacA m1*, *babA*, and *dupA* form a group in which the co-occurrence between all gene pairs is statistically significant. The only pair in this group that did not exhibit statistically significant co-occurrence was *cagA*–*babA*. No association was found between *oipA* or *iceA* and any of the other genes (*p* > 0.05). Detailed results are summarized in Supplementary Table S5.

### 3.4. The Relationship Between Virulence Factors and Sex

After confirming the significant association between males and *H. pylori* infection, we tested which of this bacterium’s genes are sex-linked and found that the presence of the *cagA*, *cagE*, and *virB11* genes as well as infection by multiple strains are significantly associated with males (*p* = 0.001, *p* = 0.003, *p* =0.014, and *p* =0.008, respectively, Supplementary Table S6).

### 3.5. Association of H. pylori Genes with Histopathological Outcomes

**Figure 3:**
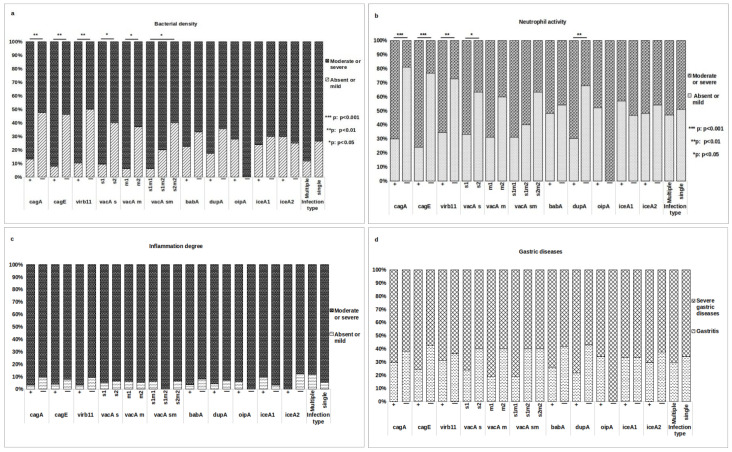
Relationship between *H. pylori* genes and histopathological manifestations. Association tests were conducted to assess the relationship between various virulence genes and histopathological findings, including (**a**) bacterial density, (**b**) neutrophil activity, (**c**) degree of inflammation, and (**d**) gastric disease. The association test was performed on two groups of patients: those infected with a single strain and having a complete genotype (51/70 patients) and the overall group of patients classified by type of infection (70 patients). Variables were compared using the Chi-squared or Fisher exact test. (*) denotes statistically significant results.

A bivariate analysis was conducted to investigate the association between each *H. pylori* gene and histopathological findings. The presence of *cagA*, *cagE*, *virB11*, *vacA s1*, *vacA m1*, and *vacA s1m1* exhibited a significant association with moderate or severe bacterial density (*p* = 0.007, *p* = 0.002, *p* = 0.002, *p* = 0.016, *p* = 0.039, and *p* = 0.047, respectively, Figure 3a). Additionally, the presence of *cagA*, *cagE*, *virB11*, *vacA s1*, and *dupA* showed a positive correlation with moderate/severe neutrophil activity (*p* < 0.001, *p* < 0.001, *p* = 0.007, *p* = 0.035, and *p* = 0.008, respectively, Figure 3b). No significant association was found between these genes and the degree of inflammation or severity of gastric disease (Figure 3c,d). Notably, the *babA*, *oipA*, and *iceA* genes were not associated with any of the histopathological parameters.

We proceeded to categorize patients based on MCA results and the presence or absence of five virulence genes: *cagA*, *cagE*, *virB11*, *vacA s1m1*, and *babA*. Subsequently, patients were classified according to combinations of these genes into three groups: [*cagA* (+), *cagE* (+), *virB11* (+), *vacA s1m1*, *babA* (+)]; [*cagA* (-), *cagE* (-), *virB11* (-), *vacA s2m2*, *babA* (-)]; and [*cagA* (-), *cagE* (-), *virB11* (-), *vacA s2m2*, *babA* (+)]. A notable positive association was observed between the combination [*cagA* (+), *cagE* (+), *virB11* (+), *vacA s1m1*, *babA* (+)] and severe gastric disease (OR = 8.73; 95% CI: 1.03–74.12, *p* = 0.038). Conversely, the combination [*cagA* (-), *cagE* (-), *virB11* (-), *vacA s2m2*, *babA* (-)] exhibited a negative correlation with moderate/severe neutrophil activity (OR = 0.16, 95% CI: 0.03–0.86, *p* = 0.021). No significant association was identified between the combination [*cagA* (-), *cagE* (-), *virB11* (-), *vacA s2m2*, *babA* (+)] and the studied histopathological parameters (Table 1).

We stratified the various gastric diseases to discern the specific combination(s) associated with each severe gastric disease. The results demonstrate a substantial association between the combination [*cagA* (+), *cagE* (+), *virB11* (+), *vacA s1m1*, *babA* (+)] and gastric cancer (OR = 11.10, 95% CI: 1.04–118.57, *p* = 0.046). Furthermore, gastric atrophy is associated with the combination [*cagA* (-), *cagE* (-), *virB11* (-), *vacA s2m2*, *babA* (+)] (OR = 10.25, 95% CI: 1.53–68.62, *p* = 0.028). No significant association was found between intestinal metaplasia, follicular gastritis, and the investigated combinations of genes (Table 2).

For histopathological parameters, multivariate analysis was performed, adjusting for risk factors, including *H. pylori* virulence genes, demographic factors and bacterial density (Supplementary Table S7). Concerning moderate or severe neutrophil activity, the final model identified the *cagE* gene as the exclusive predictive factor (OR = 4.99, 95% CI: 1.18–21.09; *p* = 0.03) for this parameter. Despite significant associations observed in bivariate analysis with male subjects (OR = 5.77, 95% CI: 1.72–19.29, *p* = 0.003) and moderate or severe bacterial load (OR = 9.86, 95% CI: 1.92–50.70, *p* = 0.002), these variables were not retained in the multivariate analysis. Additionally, the final model highlighted that patients infected with *H. pylori* strains carrying the gene combination [*cagA* (+), *cagE* (+), *virB11* (+), *vacA s1m1*, *babA* (+)] faced a significantly higher risk of developing severe gastric disease, particularly gastric cancer (OR = 11.10, 95% CI: 1.04–118.57, *p* = 0.046). Furthermore, a combination of genes [*cagA* (-), *cagE* (-), *virB11* (-), *vacA s2m2*, *babA* (+)] was identified as a risk factor for gastric atrophy (OR = 10.25, 95% CI: 1.53–68.62, *p* = 0.016). In contrast, the development of follicular gastritis showed no association with *H. pylori* virulence factors but was correlated with a moderate or severe bacterial load (OR = 15.38, 95% CI: 1.64–144.40, *p* = 0.017) and age ≤ 40 (OR = 5.87, 95% CI: 1.46–23.58, *p* = 0.013) (Table 3).

## 4. Discussion

*H. pylori* colonize the gastric mucosa of more than half the world’s population and could lead to the development of severe gastric disease. The outcome of *H. pylori* infection depends on multiple factors, including strain genotype, providing opportunities for the development of prognosis biomarkers of gastric disease severity. However, only a few populations have been subject to biomarker testing for gastric disease using multiple virulent genes. To the best of our knowledge, our study is the first investigation in North Africa to simultaneously examine the correlation between multiple *H. pylori* virulence genes (*cagA*, *cagE*, *virB11*, *vacA*, *dupA*, *babA*, *iceA*, and *oipA*) and histopathological outcomes. This study provides a foundation for future biomarker research.

The *H. pylori* infection rate in our study (58.3%) is similar to that reported in northeastern Morocco in 2020 by El Khadir and colleagues (60.9%) [25]. Once in the gastric lumen, *H. pylori* colonize epithelial cells, and increased bacterial density indicates successful adaptation. Infection triggers a host immune response by recruiting inflammatory cells (neutrophils, monocytes, lymphocytes, etc.), which eventually induce gastritis and potentially lead to severe gastric disease [3]. Our findings, in line with previous studies [12,25,26], provide evidence for the association of *H. pylori* infection with moderate to severe levels of neutrophil activity and inflammation, as well as with severe gastric disease.

*H. pylori* is a genetically diversified organism, and its genotypes are not equally distributed worldwide [21]. Our findings of the *oipA* adhesin gene prevalence, are consistent with studies conducted in Tunisia, korea, and Costa Rica [27,28,29], where a high prevalence (>90%) was reported. For the *babA* adhesin gene, prevalence (55.7%) was close to that of Turkish and Iranian populations [30,31], but it was significantly lower than those reported in Korea and Venezuela [27,32]. The predominance of *iceA2* subtype in our population is in agreement with reports from American countries [8,28], unlike Turkey, China and Saudi Arabia [8,30,33] and where *iceA1* is the most prevalent. The occurrence of the *dupA* gene was 31% in Asian countries and 64.1% in Western countries [7]. However, there is limited information on its prevalence in African countries. The detection rate of the *dupA* gene in our study (54.3%) aligns with the findings reported in populations from South Africa and Sudan [34,35].

The *vacA* gene, *vacA s2m2* combination is the most frequently observed, followed by *vacA s1m1* and *vacA s1m2*. This pattern is consistent with previous studies in Morocco and North African countries [25,29,36]. However, it differs from results observed in South Africa, Sudan, and Brazil, where the toxigenic *vacA s1m1* type is more frequent [4,34,35]. The frequency of multiple infections was 24.3% in our study, which is consistent with the results of Sheu’s team (23.3%) and Chih-Ho Lai (28.6%) [37], but higher than the rate reported in a previous study from northeastern Morocco (14.2%) [13]. This difference may be attributed to the fact that we examined two polymorphic genes (*vacA* and *iceA*) instead of one.

The prevalence of *cagPAI* genes, including *cagA* (62.9%), *virB11* (60%), and *cagE* (51.4%), in our population is comparable to findings from Brazilian studies [21], but notably lower than in Thai and Korean populations [38]. The presence of intact *cagPAI* in a majority of the East Asian population partially accounts for the higher incidence of gastric cancer in these countries.

Independently of histopathological findings, the analysis of the relationship between genes revealed a positive association among *cagA*, *cagE*, *virB11*, *vacA s1*, *vacA m1*, *dupA*, and *babA*, with the exception of the *cagA*-*babA* pair. This result is consistent with several studies [21,30,34,39] and suggests that specific gene combinations are under selective pressure, despite their non-proximal locations in the *H. pylori* genome.

Interestingly, we found that infection by *H. pylori* containing the *cagPAI* genes (*cagA*, *cagE*, or *virB11*), as well as infection with multiple strains, was significantly higher in males than in females. It is noteworthy that among the male patients infected with multiple strains, 71.43% had at least one of the studied *cagPAI* genes, a finding that could partially explain this association between multiple infections and the male sex. This male susceptibility to *H. pylori* infection could be attributed to the immunosuppressive effects of testosterone, while the female hormones (estradiol and progesterone) have been shown to possess bacteriostatic and bactericidal effects against this bacterium [40,41]. Taken together, these factors may partially account for the higher incidence of gastric cancer in males [42]. We note that few inconsistent studies have examined the relationship between *cagA* or *cagE* and sex, and to our knowledge, there is no prior data on the association of the *virB11* gene with the sex of the patient. The lifestyle of males and their exposure to more virulent *H. pylori* strains in the geographic location samples could explain, in part, the association between these genes and males. However, further research is needed to fully understand the complex interplay between these factors.

In a step to understand the link between *H. pylori*’s virulence genes and the various stages of pathogenesis, we conducted bivariate and multivariate analyses to explore the connection between these genes, both individually and collectively, and histopathological outcomes. No statistical association was found between the *iceA* or *oipA* genes and histopathological parameters in our analysis. The correlation between the *iceA* gene and clinical outcome has been investigated in several studies, revealing geographical dependence [28,29,33]. The association of the *oipA* gene with severe clinical outcomes is still debated and relies on the “on” status of the gene [6,27]. Therefore, in regions where the *oipA* gene is prevalent, such as ours, sequencing is required to determine its “on” or “off” status.

Our results, in line with numerous studies, confirm the correlation between colonization and the individual presence of the *cagA*, *cagE*, *virB11*, *vacA s1*, *vacA m1* and *vacA s1m1* genes [23,43]. We did not observe any significant correlation between *babA* and bacterial density. Nevertheless, *H. pylori* colonization does not depend solely on the BabA-Leb interaction, as it may also be mediated by the HpaA and NapA adhesins, or by other adhesins that we have not studied here [11].

Infection induces damage to gastric cells by two interconnected mechanisms: direct interaction with epithelial cells or via secretion of virulence factors that trigger an inflammatory response within epithelial cells. This response entails the activation of various pro-inflammatory cytokines and chemokines, leading to the mobilization of inflammatory cells for pathogen eradication. Our results show a significant correlation between the presence of *cagA*, *cagE*, *virB11*, *vacA s1*, or *dupA* and moderate/severe neutrophil activity. Remarkably, multivariate analysis identified the *cagE* gene as a robust predictor of increased neutrophil activity. This result reinforces the idea that the induction of IL-8 secretion, an important neutrophil chemotactic, is CagE dependent [14]. Notably, the absence of CagE results in the complete loss of the T4SS cytoplasmic complex, including structures containing Cagβ and VirB11 (Cagα), thus impeding CagA translocation [15].

Despite the presence of a significant association between *H. pylori* infection and a substantial level of inflammation, intriguingly, no significant correlation was observed between the examined genes and the degree of inflammation. This result diverges from studies reported by other countries [23,35,44,45]. This discrepancy is likely attributable to factors beyond bacterial genotype, including high bacterial density or host and environmental factors.

According to our above-mentioned findings, the *dupA* gene is implicated in increased neutrophil activity. A meta-analysis and systematic review conducted by Seiji Shiota and colleagues provide evidence of its association with the risk of developing duodenal ulcers [7]. Unlike other virulence factors of *H. pylori*, *dupA* exerts a protective effect against severe gastric diseases, such as gastric atrophy, intestinal metaplasia, and gastric cancer, by minimizing disruption of the gastric microbiome [9,46]. With the aim of identifying a wide range of genes for severe gastric disease, we excluded the *dupA* gene from gene combination analyses to maintain the predictive efficacy of other genes in assessing disease progression.

The results observed in our multivariate analysis of gastric atrophy and gastric cancer may be explained in part by the fact that the functional role of the BabA adhesin could expand to inducing DNA double-strand breaks independently of VacA and CagPAI [47]. Prolonged infection and the presence of strains harboring the *babA* gene, in addition to the *vacA* and *cag*PAI genes, can intensify and accumulate these breaks through the production of VacA and CagA toxins. Simultaneously, this process disrupts apoptotic and DNA repair mechanisms, promoting genome instability and facilitating the malignant transformation of cells [47,48].

Regarding the development of follicular gastritis, multivariate analysis revealed that age ≤ 40 years (OR = 5.87, *p* = 0.013) and moderate or severe bacterial density (OR = 15.38, *p* = 0.017) are predictive factors for its occurrence. This association is probably attributed to the persistent presence of *H. pylori* from early childhood, which stimulates the production of B lymphocytes and leads to the formation of lymphoid follicles. Although these follicles are unable to eliminate bacteria, they subsequently form the basic tissue in which MALT lymphoma can develop [1].

Our research has certain limitations that should be noted. Firstly, the results may not be fully representative of the diverse urban and rural populations across various southern regions of Morocco. Secondly, the prevalence of multiple infections, which is slightly high, may mask other genetic combinations potentially associated with severe gastric diseases. Thirdly, the limited sample size in the stages of severe gastric disease necessitates further sample collection. Despite these limitations, it is essential to note that all statistical tests have been validated. Our findings provide valuable insights into the risk factors associated with specific severe gastric diseases, serving as a foundation for the development of screening and prevention strategies.

## 5. Conclusions

In conclusion, the presence or absence of genes in specific combinations of five genes [*cagA*, *cagE*, *virB11*, *vacA*, *babA*] holds predictive value for severe gastric diseases, such as gastric atrophy and gastric cancer. Additionally, age and bacterial load proved useful in predicting follicular gastritis. Furthermore, the *cagE* gene has emerged as a biomarker associated with heightened neutrophil activity. These results provide additional pieces in the puzzle of *H. pylori* and gastric disease.

## Figures and Tables

**Table 1:** Association of *H. pylori* gene combinations with histopathological outcomes.

		Bacterial Density *n* (%)		Neutrophil Activity *n* (%)		Inflammation Degree *n* (%)		Gastric Diseases *n* (%)	
Combinations		Absent or Mild	Moderate or Severe	Absent or Mild	Moderate or Severe	Absent or Mild	Moderate or Severe	Gastritis	Severe Gastric Diseases
***cagA* (+), *cagE* (+), *virB11* (+), *vacA s1m1*, *babA* (+)**	(+)	1/13 (7.7)	12/13 (92.3)	4/13 (30.8)	9/13 (69.2)	1/13 (7.7)	12/13 (92.3)	1/13 (7.7)	12/13 (92.3)
	(−)	13/38 (34.2)	25/38 (65.8)	22/38 (57.9)	16/38 (42.1)	2/38 (5.3)	36/38 (94.7)	16/38 (42.1)	22/38 (57.9)
***p*-value**		0.082		0.091		1		0.038 *	
**ORᵘ (CI 95%)**		**-**		**-**		**-**		**8.73 (1.03–74.12)**	
***cagA* (** **−** **), *cagE* (** **−** **) *virB11* (** **−** **), *vacA s2m2*, *babA* (** **−** **)**	(+)	5/11 (45.5)	6/11 (54.5)	9/11 (81.8)	2/11 (18.2)	2/11 (18.2)	9/11 (81.8)	5/11 (45.5)	6/11 (54.5)
	(−)	9/40 (22.5)	31/40 (77.5)	17/40 (42.5)	23/40 (57.5)	1/40 (2.5)	39/40 (97.5)	12/40 (30)	28/40 (70)
***p*-value**		0.148		0.021 *		0.114		0.472	
**ORᵘ (CI 95%)**		**-**		**0.16 (0.03–0.86)**		**-**		**-**	
***cagA* (** **−** **), *cagE* (** **−** **), *virB11* (** **−** **), *vacA s2m2*, *babA* (+)**	(+)	3/7 (42.9)	4/7 (57.1)	5/7 (71.4)	2/7 (28.6)	0/7 (0.0)	7/7 (100)	2/7 (28.6)	5/7 (71.4)
	(−)	11/44 (25)	33/44 (75)	21/44 (47.7)	23/44 (52.3)	3/44 (6.8)	41/44 (93.2)	15/44 (34.1)	29/44 (65.9)
***p*-value**		0.376		0.419		1		1	
**ORᵘ (CI 95%)**		**-**		**-**		**-**		**-**	

Bold values and (*) indicate statistically significant results. Variables were compared using the Chi-squared or Fisher exact test. ORᵘ: unadjusted OR. The presence of an individual virulence gene is denoted by ‘+’, while its absence is indicated by ‘–’. Similarly, for haplotypes or gene combinations, presence is marked by ‘+’ and absence by ‘–’ in front of the respective combination.

**Table 2:** Stratification of severe gastric disease and its association with *H. pylori* gene combinations.

		Follicular Gastritis *n* (%)		Gastric Atrophy *n* (%)		Intestinal Metaplasia *n* (%)		Gastric Cancer *n* (%)	
Combinations		(+)	(−)	(+)	(−)	(+)	(−)	(+)	(−)
***cagA* (+), *cagE* (+), *virB11* (+), vacA *s1m1*, *babA* (+)**	(+)	6/13 (46.2)	7/13 (53.8)	0/13 (0.0)	13/13 (100)	2/13 (15.4)	11/13 (84.6)	3/13 (23.1)	10/13 (76.9)
	(−)	11/38 (28.9)	27/38 (71.1)	6/38 (15.8)	32/38 (84.2)	1/38 (2.6)	37/38 (97.4)	1/38 (2.6)	37/38 (97.4)
***p*-value**		0.315		0.318		0.156		0.046 *	
**ORᵘ (CI 95%)**		**-**		**-**		**-**		**11.10 (1.04–118.57)**	
***cagA* (−), *cagE* (−), *virB11* (−), *vacA s2m2*, *babA* (−)**	(+)	4/11 (36.4)	7/11 (63.6)	1/11 (9.1)	10/11 (90.9)	0/11 (0.0)	11/11 (100)	0/11 (0.0)	11/11 (100)
	(−)	13/40 (32.5)	27/40 (67.5)	5/40 (12.5)	35/40 (87.5)	3/40 (7.5)	37/40 (92.5)	4/40 (10)	36/40 (90)
***p*-value**		1		1		1		0.565	
**ORᵘ (CI 95%)**		**-**		**-**		**-**		**-**	
***cagA* (−), *cagE* (−), *virB11* (−), *vacA s2m2*, *babA* (+)**	(+)	2/7 (28.6)	5/7 (71.4)	3/7 (42.9)	4/7 (57.1)	0/7 (0.0)	7/7 (100)	0/7 (0.0)	7/7 (100)
	(−)	15/44 (34.1)	29/44 (65.9)	3/44 (6.8)	41/44 (93.2)	3/44 (6.8)	41/44 (93.2)	4/44 (9.1)	40/44 (90.9)
***p*-value**		1		0.028 *		1		1	
**ORᵘ (CI 95%)**		**-**		**10.25 (1.53–68.62)**		**-**		**-**	

Bold values and (*) indicate statistically significant results. Variables were compared using Fisher exact test. ORᵘ: unadjusted OR. The presence of an individual virulence gene is denoted by ‘+’, while its absence is indicated by ‘–’. Similarly, for haplotypes or gene combinations, presence is marked by ‘+’ and absence by ‘–’ in front of the respective combination.

**Table 3:** Significant risk factors associated with histopathological outcomes after adjusting for genotype, demographic factors, and bacterial density.

		Histopathological Outcomes		Bivariate Analysis		Multivariate Analysis	
Risk Factors				*p*-Value	ORᵘ (CI 95%)	*p*-Value	ORª (CI 95%)
		**Neutrophil activity *n* (%)**					
		Absent or mild	Moderate or severe				
*cagE*	(+)	6 (24)	19 (76)	*p* < 0.001 *	**10.56 (2.89–38.50)**	0.03 *	**4.99 (1.18–21.09)**
	(−)	20 (76.9)	6 (23.1)		ref		ref
		**Gastric diseases *n* (%)**					
		Gastritis	Severe gastric diseases				
*cagA* (+), *cagE* (+), *virB11* (+), *vacA s1m1*, *babA* (+)	(+)	1 (7.7)	12 (92.3)	0.038 *	**8.73 (1.03–74.12)**	0.047 *	**8.73 (1.03–74.12)**
	(−)	16 (42.1)	22 (57.9)		ref		ref
		**Follicular gastritis *n* (%)**					
		(+)	(**−**)				
Age	≤40	12 (48)	13 (52)	0.029 *	**3.88 (1.11–13.55)**	0.013 *	**5.87 (1.46–23.58)**
	>40	5 (19.2)	21 (80.8)		ref		ref
Bacterial density	Moderate or severe	16 (43.2)	21 (56.8)	0.019 *	**9.90 (1.17–83.80)**	0.017 *	**15.38 (1.64–144.40)**
	Absent or mild	1 (7.1)	13 (92.9)		ref		ref
		**Gastric atrophy *n* (%)**					
		(+)	(−)				
*cagA* (**−**), *cagE* (**−**), *virB11* (**−**), *vacA s2m2*, *babA* (+)	(+)	3 (42.9)	4 (57.1)	0.028 *	**10.25 (1.53–68.62)**	0.016 *	**10.25 (1.53–68.62)**
	(−)	3 (6.8)	41 (93.2)		ref		ref
		**Gastric cancer *n* (%)**					
		(+)	(−)				
*cagA* (+), *cagE* (+), *virB11* (+), *vacA s1m1*, *babA* (+)	(+)	3 (23.1)	10 (76.9)	0.046 *	**11.10 (1.04–118.57)**	0.046 *	**11.10 (1.04–118.57)**
	(−)	1 (2.6)	37 (97.4)		ref		ref

ORᵘ: unadjusted OR, ORª: adjusted OR, ref: reference group. Bold values and (*) indicate statistically significant results. The presence of an individual virulence gene is denoted by ‘+’, while its absence is indicated by ‘–’. Similarly, for haplotypes or gene combinations, presence is marked by ‘+’ and absence by ‘–’ in front of the respective combination.

## Data Availability

The original contributions presented in this study are included in the article/Supplementary Materials. Further inquiries can be directed to the corresponding author.

## Supplementary Materials

The following supporting information can be downloaded at https://www.mdpi.com/article/10.3390/pathogens14030279/s1: Table S1: General characteristics of the 120 patients studied from the population of southern Morocco; Table S2: Primer sequences and PCR conditions; Table S3: Association of demographic characteristics of the population of southern Morocco with H. pylori infection status; Table S4: Distribution of H. pylori genotypes in infected patients from southern Morocco; Table S5: Relationship between *cagA*, *cagE*, *virb11*, *vacA*, *babA*, *dupA*, *iceA*, and *oipA* genes; Table S6: Relationship between virulence factors and gender in patients infected with *H. pylori* in southern Morocco; Table S7: Comprehensive multivariate analysis of factors associated with histopathological outcomes from the population of southern Morocco adjusted for *H. pylori* genotype, demographic factors, and bacterial density after excluding the collinear factors.

## Abbreviations

The following abbreviation is used in this manuscript:

MCA	multiple correspondence analysis
